# Nucleotide Second Messenger-Based Signaling in Extreme Acidophiles of the *Acidithiobacillus* Species Complex: Partition Between the Core and Variable Gene Complements

**DOI:** 10.3389/fmicb.2019.00381

**Published:** 2019-03-07

**Authors:** Ana Moya-Beltrán, Camila Rojas-Villalobos, Mauricio Díaz, Nicolás Guiliani, Raquel Quatrini, Matías Castro

**Affiliations:** ^1^Microbial Ecophysiology Laboratory, Fundación Ciencia & Vida, Santiago, Chile; ^2^Facultad de Ciencias de la Vida, Universidad Andrés Bello, Santiago, Chile; ^3^Millennium Nucleus in the Biology of Intestinal Microbiota, Santiago, Chile; ^4^Laboratorio de Comunicación Bacteriana, Departamento de Biología, Facultad de Ciencias, Universidad de Chile, Santiago, Chile

**Keywords:** *Acidithiobacillia*, biofilm, c-di-GMP, extremophile, nucleotide second messenger, signal transduction, (p)ppGpp, cAMP

## Abstract

Cyclic and linear nucleotides are key elements of the signal transduction networks linking perception of the environment to specific cellular behavior of prokaryotes. These molecular mechanisms are particularly important in bacteria exposed to different, and frequently simultaneous, types of extreme conditions. This is the case in acidithiobacilli, a group of extremophilic bacteria thriving in highly acidic biotopes, that must also cope with significant variations in temperature, osmotic potentials and concentrations of various transition metals and metalloids. Environmental cues sensed by bacteria are transduced into differential levels of nucleotides acting as intracellular second messengers, promoting the activation or inhibition of target components and eliciting different output phenotypes. Cyclic (c) di-GMP, one of the most common bacterial second messengers, plays a key role in lifestyle changes in many bacteria, including acidithiobacilli. The presence of functional c-di-GMP-dependent signal transduction pathways in representative strains of the best-known linages of this species complex has been reported. However, a comprehensive panorama of the c-di-GMP modulated networks, the cognate input signals and output responses, are still missing for this group of extremophiles. Moreover, little fundamental understanding has been gathered for other nucleotides acting as second messengers. Taking advantage of the increasing number of sequenced genomes of the taxon, here we address the challenge of disentangling the nucleotide-driven signal transduction pathways in this group of polyextremophiles using comparative genomic tools and strategies. Results indicate that the acidithiobacilli possess all the genetic elements required to establish functional transduction pathways based in three different nucleotide-second messengers: (p)ppGpp, cyclic AMP (cAMP), and c-di-GMP. The elements related with the metabolism and transduction of (p)ppGpp and cAMP appear highly conserved, integrating signals related with nutrient starvation and polyphosphate metabolism, respectively. In contrast, c-di-GMP networks appear diverse and complex, differing both at the species and strain levels. Molecular elements of c-di-GMP metabolism and transduction were mostly found scattered along the flexible genome of the acidithiobacilli, allowing the identification of probable control modules that could be critical for substrate colonization, biofilm development and intercellular interactions. These may ultimately convey increased endurance to environmental stress and increased potential for gene sharing and adaptation to changing conditions.

## Introduction

*Acidithiobacillus* are chemolithoautotrophic Gram-negative bacteria that thrive in environments with few nutrients, high concentrations of heavy metals and extreme acidity. They obtain energy from the oxidation of reduced inorganic sulfur compounds (RISCs), including many metal sulfides, one of the most abundant mineral classes on earth (Rohwerder and Sand, 2007). Bio-oxidation of metal sulfides allows the release of metal cations from minerals into solution and the production of sulfuric acid as a by-product. Therefore, the acidithiobacilli not only tolerate a wide range of metal ions and low pH (Dopson et al., 2003), but also are key players in promoting these extreme conditions.

*Acidithiobacillus* species have been the focus of research in areas as diverse as astrobiology (González-Toril et al., 2005) and nanotechnology (Ulloa et al., 2018). However, it is in the context of biomining applications and associated acid mine drainage phenomena that they have been more thoroughly studied. Microbial oxidation of metal sulfides has been used by the mining industry as a biotechnological process for metal extraction, allowing the recovery of economically relevant elements such as cobalt, copper, nickel, uranium, and zinc (Harrison, 2016). On the other hand, microbial leaching activity promotes the formation of highly contaminating metal loaded acidic waters that permeate into waterways generating acid rock or acid mine drainages (ARD/AMD) both in mining sites and in naturally exposed ore deposits, acidic rivers, geothermal sites and caves, impacting enormously human life and wildlife (Johnson and Hallberg, 2005).

Despite common characteristics, the genus *Acidithiobacillus* is a heterogeneous group of bacteria, currently consisting of seven species: *A. thiooxidans* (Waksman and Joffe, 1922)*, A. ferrooxidans* (Temple and Colmer, 1951), *A. albertensis* (Bryant et al., 1983), *A. caldus* (Hallberg and Lindström, 1994)*, A. ferrivorans* (Hallberg et al., 2010), *A. ferridurans* (Hedrich and Johnson, 2013), and *A. ferriphilus* (Falagán and Johnson, 2016). The most notable differences among these include the capability to oxidize ferrous to ferric iron (*A. ferrooxidans*, *A. ferrivorans*, *A. ferridurans*, and *A. ferriphilus*); the presence/absence of flagella-based motility (Valdés et al., 2008; Castro et al., 2017); and the growth temperature ranges. While most of acidithiobacilli are mesophilic (∼30°C), the psychrotolerant *A. ferrivorans* and the moderate thermophlic *A. caldus* expand the temperature niche of this taxon, the former by being able to grow at 4°C, and the latter withstanding 45°C. These differences influence substrate colonization and dominance by the diverse acidithiobacilli in different micro-niches in natural environments as well as in bioleaching operations. Since bio-oxidation activity depends largely on the physiological state of the cell, the understanding of molecular mechanisms that allow the bacteria to sense and respond to their challenging environment becomes relevant.

Different bacteria use linear and cyclic nucleotides as second messengers to regulate diverse cellular processes in response to environmental cues. Among the most important ones are cyclic adenosine 3′,5′-monophosphate (cAMP) (McDonough and Rodriguez, 2011), cyclic guanosine 3′,5′-monophosphate (cGMP) (Marden et al., 2011), cyclic dimeric guanosine 3′,5′-monophosphate (c-di-GMP) (Römling et al., 2013), cyclic dimeric adenosine 3′,5′-monophosphate (c-di-AMP) (Corrigan and Gründling, 2013), guanosine 3′,5′-bispyrophosphate (ppGpp) and guanosine 3′-diphosphate, 5′-triphosphate (pppGpp) (Hauryliuk et al., 2015). Despite the structural diversity of these nucleotides, their signaling pathways work in analogous ways. In response to environmental signals (first messenger), different enzymatic activities catalyze the synthesis or the degradation of specific nucleotide second messengers, amplifying the signal. Nucleotide levels are mainly transduced by effector proteins, which directly bind the nucleotide and interact with target components promoting diverse responses. Synthesis and degradation enzymes together with effectors and target components that work in concerted action to produce a particular output phenotype, through a common pool of the nucleotide molecules, constitute a second-messenger control module (Hengge, 2009). These control modules show important differences in terms of the nature of detected signal and the range of the output response.

Cyclic AMP (cAMP) and (p)ppGpp have been studied for decades in the context of nutrient deprivation in *E. coli*. In this neutrophilic bacterium, amino acid deficit promotes the “stringent response,” a series of physiological changes driven by an increase of intracellular levels of (p)ppGpp. Acting at transcriptional and post-traductional levels, (p)ppGpp regulates biosynthesis of amino acid and ribosomal proteins (Potrykus and Cashel, 2008) and represses nucleotide metabolism (Gallant et al., 1971; Hochstadt-Ozer and Cashel, 1972). Furthermore, in response to low energy charge (low levels of ATP), *E. coli* increases the intracellular cAMP concentration, promoting catabolism and inhibiting anabolism through gene regulation (Narang, 2009). More recently, work on the α-proteobacterium *Rhodospirillum centenum* demonstrated the role of cGMP in cyst development, metabolically dormant cells able to survive environmental stresses such as desiccation and nutrient starvation (Marden et al., 2011). Cyclic-di-AMP has been implicated in diverse essential cellular processes in bacteria (mainly in Gram-positive), including cell wall and membrane homeostasis, regulation of potassium ion channels, DNA damage repair, and sporulation (Commichau et al., 2018), although the triggering environmental stimuli are less well-characterized. Cyclic-di-GMP is the most common bacterial second messenger, being widespread in most bacterial phyla, in which it controls a variety of processes related with lifestyle and the switch from the individual motile state to the multicellular biofilm state (Jenal et al., 2017).

These signal transduction pathways are particularly important in bacteria exposed to different, and frequently simultaneous, types of extreme conditions. This is the case of the acidithiobacilli, which in addition to enduring extreme acidic conditions must cope with significant variations in temperature, osmotic potentials and concentrations of various transition metals and metalloids. Despite this fact, little is known about the scope of regulation through second messenger-driven mechanisms in this group of polyextemophiles. Recently, some components of the c-di-GMP pathway of type strains of *A. ferrooxidans* (Ruiz et al., 2012), *A. thiooxidans* (Díaz et al., 2013, 2018), and *A. caldus* (Castro et al., 2015) have been characterized. In these acidophiles, c-di-GMP signaling is related to biofilm development, a key lifestyle for substrate colonization and energy acquisition. However, as it occurs in most bacteria, the multiplicity of c-di-GMP related genes in the same microorganism hinders the determination of specific c-di-GMP control modules. Moreover, the presence and expression of multiple c-di-GMP related genes under the same conditions (Castro et al., 2015; Díaz et al., 2018) suggest the need for a spatial post-translational regulation.

Taking advantage of the increasing number of sequenced genomes of the taxon, here we address the challenge of disentangling the nucleotide-driven signal transduction pathways in this group of polyextremophiles by using a comparative genomics strategy. First, the presence, occurrence, and conservation of nucleotide second messenger related gene products in available bacterial genomes of *Acidithiobacillus* spp. were assessed. Second, to circumscribe specific c-di-GMP control modules, the distribution of metabolism-effector-target genes on discrete genetic units, such as secondary replicons and mobile genetic elements (MGEs), was analyzed. Finally, to evaluate the interconnection of predicted control/effector/target gene modules, the sub-cellular localization of c-di-GMP related proteins and the concurrence of signal-sensing associated domains were examined.

**Table 1:** Occurrence and abundance of proteins predicted to be involved in synthesis and/or degradation of nucleotide second messengers in *Acidithiobacillia*.

		(p)ppGpp		cAMP		c-di-AMP			c-di-GMP		
		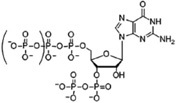		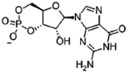		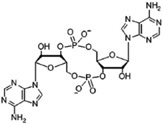			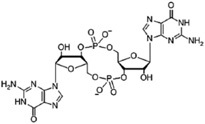		

Microorganism	No. of genomes	Synthetase/hydrolase	Hydrolase	NC class IV	Hydrolase	DAC	PDE	DGC/PDE	DGC	PDE	PDE
		SYN/HYD	HYD	CYTH	CpdA	DisA_N	DHH	GGDEF-EAL	GGDEF	EAL	HD_GYP
*T. tepidarius*	1	1	-	1	2	-	1	13	9	3	4
*A. caldus*	7	1	-	1	3-5	-	1-2	4-10	7-9	2-4	-
*A. albertensis*	1	1	-	1	2	-	2	16	7	4	2
*Acidithiobacillus* sp.	2	1	-	1	3	-	2	7-13	3-5	1-2	1-3
*A. thiooxidans*	10	1	0-1	1	2-3	-	1-3	4-7	7-11	1-3	1-2
*A. ferrivorans*	6	1	0-1	1-2	1-3	-	1-2	6-21	1-4	0-1	1-2
*A. ferrooxidans*	8	1	-	1-2	2-4	-	0-2	4-8	0-1	0-1	0-1

*The numbers correspond to the range of elements found different strain of the same species.*

## Materials and Methods

### Data Collection and Formatting: Genomes and Query Profiles

**FIGURE 1:**
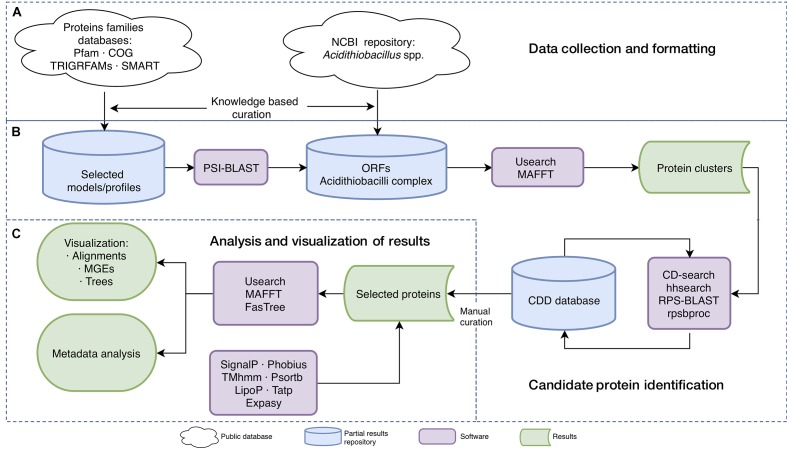
*In silico* analysis workflow. Overview of the datasets and general strategy used in the study. **(A)** Data collection and formatting. **(B)** Candidate protein identification. **(C)** Analysis and visualization of results. Symbols are shown in the bottom menu.

Complete and draft genomes sequences (chromosomes and plasmids) for *Acidithiobacillus* spp. and *Thermithiobacillus tepidarius* DSM 3134 (outgroup) were downloaded from the NCBI repository as of June 2018 and filtered based on quality and completion criteria (Figure 1). All 35 chromosomal replicons retained in the analysis encode at least one 16S rRNA gene and are >90% complete; a set of plasmids (16 in total) was also included in the filtered data repository (Supplementary Table S1). Completeness was assessed as in Raes et al. (2007). Multiple sequence alignments of the protein families of interest to be used as queries in the identification of conserved domains in candidate protein sequences were downloaded from the following domain models/profiles databases: Pfam (Finn et al., 2016), COG (Tatusov et al., 2000), TIGRFAMs (Haft et al., 2013), SMART (Letunic and Bork, 2018) and clusterized to remove redundancy (see below). The full list of models/profiles used in this study can be found in Supplementary Table S2.

### Candidate Proteins Identification: Gene Annotation and Analysis

ORF prediction was done using GeneMarkS+ software with MetageneMark_v1mod model (Zhu et al., 2010) and functional assignment and curation was done using the workflow presented in Figure 1. Briefly, orthologs of the domain models/profiles of interest (see above) were identified in each genome using PSI-BLAST (with five iterations, e-value 0.01; Altschul et al., 1997) and candidate target sequence assignments were validated against the Conserved Domain Database v.3.16 (CDD, Marchler-Bauer et al., 2017) using CD-search (Marchler-Bauer and Bryant, 2004) and hhsearch (Fidler et al., 2016) with defaults parameters (e-value < 0.00001 for CD-search and hhsearch and probability > 90% for hhsearch). Identification of specific domains in selected proteins was done with RPS-BLAST v2.2.26 versus CDD database. Elimination of overlapping motifs and redundant results were done with rpsbproc v0.11.

### Analysis and Visualization of Results

Selected proteins were clustered by identity (threshold of 50% and defaults parameters) with Usearch v1.2.22 (Edgar, 2010). Protein alignments were constructed with the MAFFT v7.123 program (Katoh and Standley, 2013). Selected clusters and proteins were re-annotated and curated manually.

The FastTree program v2.1.7 (Price et al., 2010) was used for phylogenetic analysis (WAG evolutionary model for amino acid sequences analyses; GTR evolutionary model for nucleotide sequences analyses) with bootstrap resampling (1,000 replicates). FigTree v1.4.3 (Rambaut, 2009) was used for tree visualization and manipulation.

All candidate proteins were analyzed with additional resources, including SignalP v4.0 (Petersen et al., 2011) and Phobius v1.0 (Kall et al., 2004) for signal peptide prediction; TMhmm v2.0c (Krogh et al., 2001) for transmembrane helices predictions; Psortb v3.0.5 (Gardy et al., 2003) for protein subcellular localization prediction; LipoP v1.0 for lipoproteins prediction and discrimination of lipoprotein signal peptides (Juncker et al., 2003); Tatp v1.0 for Twin-arginine signal peptide cleavage sites identification (Bendtsen et al., 2005). All software was downloaded and run locally using default parameters for gram-negative bacteria. The theoretical pI (isoelectric point) of candidate proteins was computed through the Expasy server^1^.

Complete genomes (NZ_CP005986, NC_015850, NZ_CP026328, NC_015942, NZ_LT841305, NC_011761, NC_011206) were used for identification of putative MGEs using the following programs: AlienHunter (Vernikos and Parkhill, 2006), PAI-DA (Tu and Ding, 2003), IslandPath (Hsiao et al., 2005) for genomic island identification; PhiSpy (Akhter et al., 2012) and Phage Finder (Fouts, 2006) for prophage identification; TnpPred (Riadi et al., 2012) for prediction of prokaryotic transposases; CONJscan (Guglielmini et al., 2014) for Type IV secretion systems prediction; tRNAscan (Lowe and Eddy, 1997) and Aragorn (Laslett and Canback, 2004) for search tRNA and tmRNA. All predictions were analyzed and curated manually.

Protein and genome comparisons were performed with Blastp and Blastn respectively and visualized with Artemis Comparison Tool (ACT) v1.0 (Carver et al., 2005). Figures of gene contexts and neighborhood were performed with genoPlotR (Guy et al., 2010). Genome circular visualization was performed using circos (Krzywinski et al., 2009).

## Results and Discussion

### General Overview

To reconstruct nucleotide-driven signal transduction pathways in polyextremophilic acidithiobacilli, we analyzed 51 replicons [35 chromosomes (7 closed and 28 at more than 90% completeness) and 16 plasmids] belonging to *Acidithiobacillus* species (Supplementary Table S1), comprising extreme (pH ≤ 3) to moderate (3 ≤ pH ≤ 5) acidophiles and psychrotolerant (growth at 4°C) to mesothermophiles (growth at 45°). The genomes were searched for hallmark sequences containing the signature protein domains related with the synthesis and degradation of nucleotide second messengers, as well as downstream effector proteins (Supplementary Table S2). Workflow details are summarized in Figure 1.

Synthesis functions were identified in all 35 chromosomes for every signaling nucleotide targeted, except c-di-AMP (Table 1). All genomes analyzed encode (p)ppGpp synthetases and nucleotidyl cyclases (NCs) with readily identifiable domains conferring to these enzymes specific biosynthetic activities, such as adenylyl cyclases (ACs) or diguanylate cyclases (DGCs) for the synthesis of cAMP and c-di-GMP, respectively (Table 1 and Supplementary Table S3). Consistently, hydrolases for degradation of the three nucleotides are also encoded in every *Acidithiobacillus* genome analyzed. Surprisingly, hydrolases for the degradation of c-di-AMP dinucleotide were also identified in all genomes, even if the synthesizing diadenylate cyclases (DACs) appeared to be absent. Globally, these results suggest that signaling nucleotides are relevant as second messenger molecules for bacteria thriving in acidic econiches, as they are in other environments. Each of the pathways uncovered is analyzed in further detail in the sections below and, when possible, the implications in acidithiobacilli ecophysiology are inferred.

### Guanosine Tetra/Pentaphosphate Acts as Alarmone in the Acidithiobacilli

We first assessed the presence, occurrence, and conservation of guanosine tetra and/or pentaphosphate synthesis and degradation functions in *Acidithiobacillus* spp. Without exception, every genome analyzed was found to encode a single copy of the large (p)ppGpp synthetases belonging to the RSH protein superfamily (RelA/SpoT Homolog), named after the discovery of RelA and SpoT proteins in *Escherichia coli* (Stent and Brenner, 1961; Cashel and Gallant, 1969). The acidithiobacilli’s long RSHs will be collectively referred to as SpoT_Aci_ onwards (see below).

Most γ- and β-proteobacteria bare two long RSH gene orthologs per genome (*relA* and *spoT*), arisen through a gene duplication event of an ancestral bifunctional “Rel” protein (Atkinson et al., 2011). In contrast, evaluated members of *Acidithiobacillus* genus only possess one long RSH (*spoT*) per genome. This is consistent with the recent re-classification of this acidophilic taxon as a new class (*Acidithiobacillia*), ancestral to both the γ- and β-proteobacteria (Williams and Kelly, 2013). Long RSHs are conformed by the synthesis [SYN (RelA_SpoT: pfam04607)] and hydrolysis [HYD (HD: pfam13328)] domains, generally escorted by accessory domains that regulate their activities.

The SYN domain is known to catalyze the synthesis of ppGpp and pppGpp by the addition of a pyrophosphate (Pi) group from ATP to the 3′ position of GDP and GTP, respectively. SpoT_Aci_ conserve the RXKD motif at the substrate-binding pocket of the predicted SYN domain (Supplementary Figure S1A) instead of the EXDD motif present in *relA* orthologs. This aspect suggests that the acidithiobacilli SpoT_Aci_ would prefer GTP over GDP as substrate and therefore it is predicted to mainly synthesize pppGpp over ppGpp (Sajish et al., 2009). Despite this, *Acidithiobacillus* species could still produce ppGpp linear messengers through enzymatic removal of one Pi from pppGpp. All *Acidithiobacillus* species analyzed encode the *ppx* gene (exopolyphosphatase), an ortholog of the *gppA* gene encoding for the pppGpp phosphohydrolase (Keasling et al., 1993). This is relevant since pppGpp and ppGpp bind to different target effector molecules and produce different output responses (Wang et al., 2007; Mechold et al., 2013). All SpoT_Aci_ also conserve the HDXXED motif at HYD domain needed for (p)ppGpp hydrolysis (Aravind and Koonin, 1998), and are therefore predicted as bifunctional.

In well-studied microbial models, synthesis of (p)ppGpp is activated by different environmental stresses, including temperature changes (e.g., English et al., 2011) and limitation of several nutrients such as: carbon sources (e.g., Xiao et al., 1991), phosphate (e.g., Spira et al., 1995), iron (e.g., Vinella et al., 2005), fatty acids (e.g., Battesti and Bouveret, 2006), and amino acids (e.g., Haseltine and Block, 1973). Synthesis and hydrolysis of long RSHs are controlled by additional domains that integrate these diverse signals. Identified SpoT_Aci_ accessory domains are TGS (ThrRS, GTPase, and SpoT), ZFD (zinc-finger domain), and RRM (RNA recognition motif) (HYD-SYN-TGS-ZFD-RRM configuration), which are widely conserved in the protein family. The presence and conservation of the TGS domain in SpoT_Aci_ suggests that its synthetase activity may be tiriggered by amino acid and/or fatty acid starvation (Battesti and Bouveret, 2006, 2009; Winther et al., 2018). Congruently, (p)ppGpp binds to many targets including amino acid decarboxylases (LdcI/CadA, LdcC, SpeF, SpeC, SlyA, PigR, PNPase), lipid metabolism protein (PlsB, PgsA, AccA, AccD, FabA, FabZ, GdhA, GlgC, Ppc) and nucleotide metabolism proteins (DnaG, MazG, GuaB, APT, Hpt, HisG) (Kanjee et al., 2012), most of which occur in the *Acidithiobacillia* genomes screened (data not shown).

**FIGURE 2:**
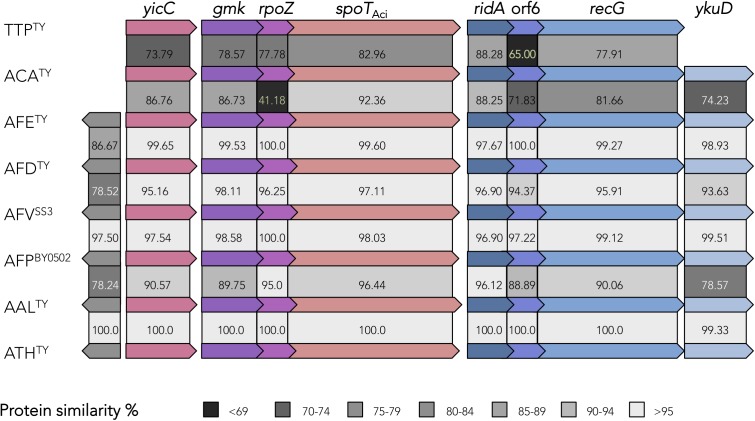
Organization and conservation of the SpoT_Aci_ gene clusters in *Acidithiobacillia* reference genomes. YicC, uncharacterized stress-induced protein (COG1561); GMK, guanylate kinase (COG0194); RpoZ, RNA polymerase, omega subunit (COG1758); SpoT, bifunctional (p)ppGpp synthetase/guanosine-3′-5′-*bis*(diphosphate) 3′pyrophosphohydrolase (COG0317); RidA, enamine deaminase (COG0251); hyp (orf6), hypothetical protein with a hybrid cluster protein-associated redox disulfide domain (TIGR03980); RecG, ATP-dependent DNA helicase (pfam13749); YkuD, murein L,D-transpeptidase catalytic domain containing protein (pfam03734) in TTP*, T. tepidarius;* ACA*, A. caldus;* AFE*, A. ferrooxidans;* AFD*, A. ferridurans;* AFV*, A. ferrivorans;* AFP*, A. ferriphilus;* AAL, *A. albertensis;* ATH*, A. thiooxidans.* Percentage of amino acid similarity is depicted.

*Acidithiobacillia* long RSH protein encoding genes occur in a highly conserved gene cluster, which resembles the *spoT* operon of *E. coli* both in structure and sequence (Figure 2). Conservation of gene context provides further clues of its functionality and expected physiological outputs. Genes immediately upstream of *spoT*_Aci_ are *gmk* and *rpoZ*. One of the best known (p)ppGpp mediated effects is its global control of gene expression, through direct binding to the RNA polymerase omega subunit (RpoZ). In this way (p)ppGpp simultaneously inhibits rRNA synthesis and the expression of ribosomal proteins (Paul et al., 2004), and activates transcription of amino acid biosynthesis genes (Paul et al., 2005). In the *Acidithiobacillia*, the RpoZ predicted proteins are well-conserved and possess the conserved MAR motif in its N-terminal for (p)ppGpp binding (Hauryliuk et al., 2015) (Supplementary Figure S1B). In turn, *gmk*, which codes for a guanylate (GMP) kinase, is a key player in *de novo* pathway for GTP synthesis catalyzing the formation of GDP from GMP (plus ATP) (Srivatsan and Wang, 2008). In other bacteria (e.g., in Firmicutes) this kinase is inhibited by (p)ppGpp promoting simultaneously the decrease of GTP levels and an accumulation of GMP, affecting the transcription of genes under GTP-dependent promotors (Kriel et al., 2012; Liu et al., 2015). The *gmk* gene occurs in just one copy per genome and its predicted protein product bares a highly conserved essential motif (K..S..RxxxR..Y..Y..E..Y..E..I..E) required for (p)ppGpp binding (Liu et al., 2015) (Supplementary Figure S1C). The co-occurrence of *gmk* and *spoT_Aci_* in the same operon and amino acidic sequence level conservation of GMK (data not shown) suggests that in *Acidithiobacillia* GMK is also inhibited by (p)ppGpp. The decrease of intracellular GTP levels may have an impact on the synthesis of others nucleotide messengers based in GTP such as c-di-GMP.

In some strains of *A. thiooxidans* (6 out of 10) and *A. ferrivorans* (2 out of 7) one copy of Small Alarmone Hydrolases (SAHs) containing just the HYD domain (Atkinson et al., 2011) were identified (Supplementary Figure S1A). Presently, there is no evidence of their functionality; yet as the SAHs of other microorganisms, it is likely that they play a role in fine-tuning sensitivity and speed of (p)ppGpp degradation (Atkinson et al., 2011).

### Functional Association Between *Phosins* and cAMP Synthesis in the *Acidithiobacillia*

A second highly conserved signal transduction pathway in *Acidithiobacillia* corresponds to the one driven by cyclic AMP (cAMP). cAMP is synthesized from ATP by cognate nucleotidyl cyclases (Supplementary Table S2). ACs are very diverse and have been grouped in six classes (I–VI), which share no sequence similarities (Bârzu and Danchin, 1994). Every *Acidithiobacillus* genome queried in this work only encodes a single putative AC belonging to class IV. No other type of ACs (classes I–III, V–VI) were detected in any of the species of the genus.

To date, only a few class IV ACs have been biochemically characterized. One of such proteins is CyaB from mesophilic bacteria *Aeromonas (Ae.) hydrophila* and *Yersinia pestis* (YpAC-IV), which are formed by a single CYTH domain (pfam01928) and exhibit thermophilic properties (Sismeiro et al., 1998; Smith et al., 2006). Residues linked to the active site of this reference protein (Iyer and Aravind, 2002; Gallagher et al., 2011) are well-conserved in the predicted CYTH domains from ACs found in the acidithiobacilli (AC_Aci_) (Supplementary Figure S1D). In *Ae. hydrophila* biochemical conditions for maximal enzymatic activity of CyaB were found to be 65°C and pH 9.5, while a second AC CyaA (class I) performs optimally at 37–45°C and pH 8.5, suggesting that cAMP is synthetized under different *in vivo* situations by either one of the enzymes (Sismeiro et al., 1998). Despite its range of temperature (4–45°C) and pH (2.5–4.5) for growth, no other type of AC beside the class IV AC_Aci_ was detected in the *Acidithiobacillia* class, suggesting that the capacity to synthetize cAMP in response to different conditions depends specifically on a single AC protein.

**FIGURE 3:**
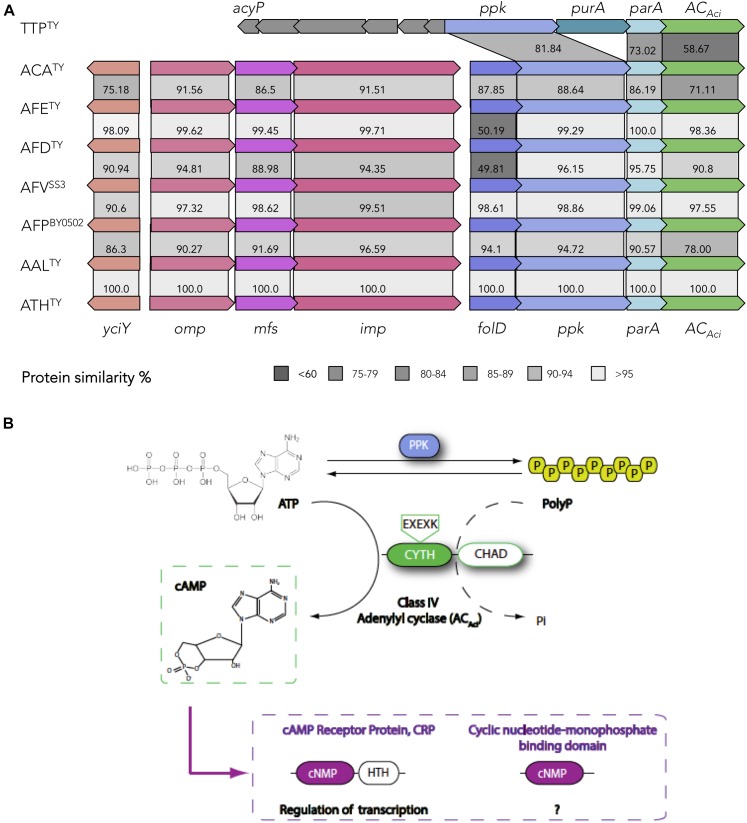
cAMP-dependent signaling in the *Acidithiobacillia* class. **(A)** Organization of the AC_Aci_ operon and conservation of the protein coding genes. Percentage of amino-acid similarity is indicated. **(B)** Model of cAMP-dependent signaling in the acidithiobacilli. ATP serves as precursor in the synthesis of cAMP. Levels of ATP and PolyP are modulated by the activity of PPK and AC_Aci_. A putative alternative reaction catalyzed by AC_Aci_, the polyP degradation is indicated by a dashed arrow. cAMP synthetized by AC_Aci_ binds to cAMP receptor proteins (CRPs) and transduces the signal. Outer membrane (*omp*), periplasmic (*mfp*) and inner membrane (*imp*) subunits of the Resistance-Nodulation-Division (RND) transport; *folD*, methenyltetrahydrofolate cyclohydrolase FolD; *ppk*, polyphosphate kinase PPK1; *parA*, chromosome partitioning protein ParA.

Genomic evidence has shown that CYTH domains of many ACs co-occur in the same microbe, or in the same polypeptide, with few different domains (Iyer and Aravind, 2002). In the case of *Acidithiobacillus* AC_Aci_, a “conserved histidine alpha-helical domain” (CHAD) is present at the proteins C-terminus (Figure 3A). CHAD domain is qualified as *phosin* given its capacity to bind polyphosphate (polyP) granules (Tumlirsch and Jendrossek, 2017). Therefore, the cyclase activity of AC_Aci_ is probably regulated by the presence of polyP. PolyP is a linear polymer of 1000s of orthophosphate residues linked by phosphoanhydride bonds, involved in diverse roles in both Prokaryotes and Eukaryotes. The main enzyme involved in the biosynthesis of polyP in *Acidithiobacillus* is the *phosin* PolyP kinase (PPK), catalyzing the reversible conversion of the terminal phosphate of ATP into polyP (Orell et al., 2012). The presence of *ppk* in the immediate vicinity of the AC_Aci_ encoding gene suggests a functional association between these two proteins. A direct connection could be established by the reverse activity of PPK providing the substrate ATP to AC_Aci_ for cAMP synthesis (Figure 3B). Besides, AC_Aci_ could represent an ancient partner of PPK in PolyP metabolism, since CYTH domain (named due to its presence in enzymes acting on triphosphorylated substrates, CyaB from *Ae. hydrophila* and the human Thiamine triphosphatase) has been proposed to work as phosphoesterase through a two-metal reaction mechanism, with the AC activity being a secondary reaction (Iyer and Aravind, 2002). The cognate enzyme that liberates inorganic phosphate (Pi) from polyP hydrolysis is a polyphosphatase (PPX) (Rao et al., 2009). In *E. coli*, both *ppk* and *ppx* genes are encoded in the same operon, while in *Acidithiobacillus* spp. *ppx* forms part of the *pho* operon (Navarro et al., 2013). In the acidithiobacillli, PolyP is a key source of Pi for metal detoxification, effecting and/or facilitating the formation of metal-phosphate and metal-sulfur complexes and their elimination (Alvarez and Jerez, 2004; Navarro et al., 2013; Ulloa et al., 2018). In this context, occurrence of a conserved RND family efflux pump in the gene neighborhood of AC*_Aci_* suggests a possible implication of this pump in P-metal complexes elimination.

Another gene in the AC_Aci_ vicinity is *folD* (Figure 3A), which appears conserved in all *Acidithiobacillus* analyzed and several other gamma-proteobacteria (data not shown). This gene encodes a dual function enzyme (5,10-methylenetetrahydrofolate dehydrogenase and 5,10-methenyltetrahydrofolate cyclohydrolase) central to the folate (vitamin B9)-dependent C1 metabolism, highly conserved in all domains of life for its role in the biosynthesis of key biological building blocks (nucleic acids, amino acids, provitamines, etc.) (Shen et al., 1999). In this respect, the observed operon structure suggests that cAMP acts in the acidithiobacilli as a signal connecting the energetic status of the cell (ATP and polyP levels) with central metabolism and cell division functions to act coordinately.

The major known outcome of cAMP signaling is the regulation of gene expression by direct binding to the transcription regulator CRP (cAMP receptor protein) (Green et al., 2014). All *Acidithiobacillus* genomes analyzed encode for cAMP-receptor proteins (CRPs), with the typical domain architecture described for *E. coli* (Green et al., 2014), i.e., an N-terminal region harboring the cAMP-binding domain (cNMP_binding, pfam00027), and a C-terminal region with the DNA-binding domain (canonical helix-turn-helix motif). However, these proteins are diversified among *Acidithiobacillia* members, which encode from 1 to 9 *crp* orthologs per genome depending of the species [1 plasmid pTF53 (*A. ferridurans* ATCC 33020); 3 *Acidithiobacilllus* sp. SH; 5 *T. tepidarius;* 3-5 *A. caldus*; 4-6 *A. ferrivorans*, 4-7 *A. thiooxidans*; 2-7 *A. ferrooxidans*; 9 *A. albertensis*], being *A. caldus* and *T. tepidarius* CRPs the most divergent. Intriguingly, amino acidic sequence alignment of the *Acidithiobacillia* CRP orthologs showed that the residues involved in cAMP binding (Townsend et al., 2015) are not well-conserved [G71, E72, R82, S83, T127, S128 (Supplementary Figure S1E)] and substitutions are not conservative. This could indicate absence of function, low affinity for cAMP or divergent evolution to acquire novel functions. It is known that some variations of CRP have evolved to respond at high or low cAMP intracellular concentrations (Green et al., 2014), however further studies are required for the assignment of physiological function to acidithobacilli’s CRPs.

### Diversified Cyclic Di-GMP Signaling Pathways Occur in the Acidithiobacilli

#### C-di-GMP Metabolism

We next assessed the occurrence, diversity, and abundance of proteins predicted to be involved in cyclic diguanylate (c-di-GMP) dependent signaling in the targeted *Acidithiobacillia* genomes. This cyclic nucleotide is best known for its role as second messenger controlling the transition from motile planktonic state to the attached multicellular state achieved in biofilms (Hengge, 2009). Within biofilms, bacteria benefit from physical and physiological interactions that enhance nutrient availability, potentiate toxic metabolites removal and provide protection against a variety of environmental stresses. This response is thus particularly important in extreme acidophiles, such as the acidithiobacilli.

**FIGURE 4:**
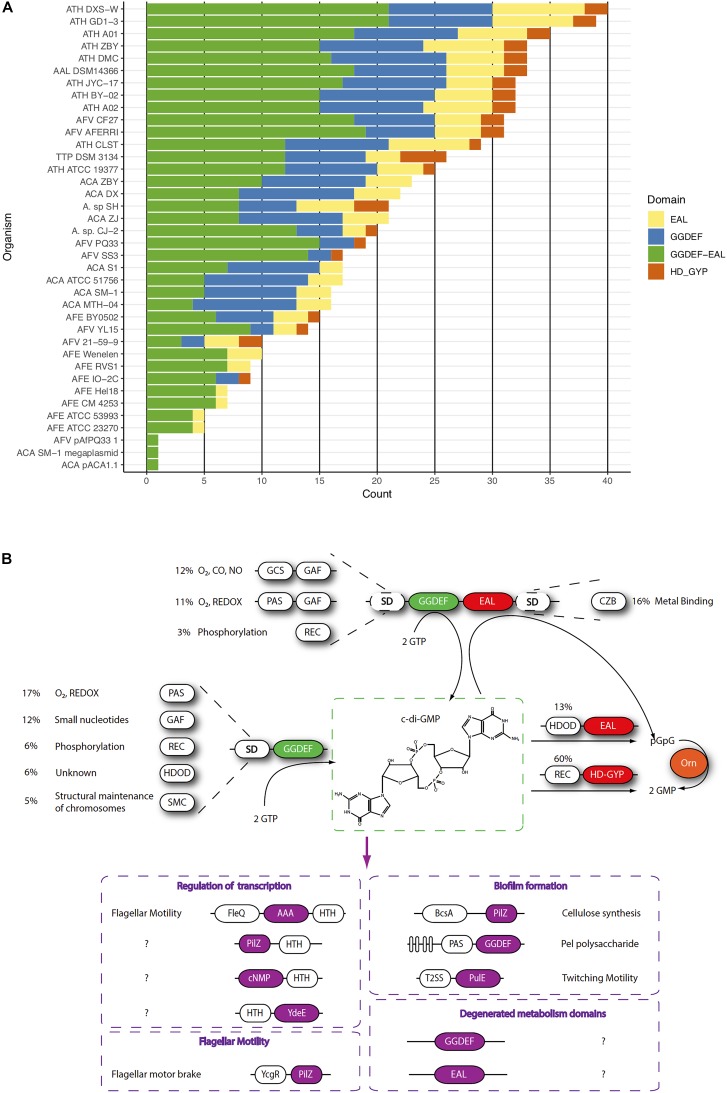
c-di-GMP-dependent pathways in the *Acidithiobacillia* class. **(A)** Frequency of c-di-GMP metabolism elements encoded in the genome of sequenced *Acidithiobacillus*. **(B)** Scheme of the signal transduction pathways) inferred from genomic information. Signals perceived by different sensor domains (SD) are transduced into the cell as differential levels of c-di-GMP through regulation of DGC and PDE activities residing on GGDEF, and EAL and HDGYP domains, respectively. Different c-di-GMP pools affect several cellular responses through diverse receptor protein families (purple). Protein domains are colored as follows: GGDEF (green), EAL and HDGYP (red), Oligoribonuclease Orn (orange).

**Table 2:** Localization signals and partner domains occurrence in the *Acidithiobacillia* class.

	GGDEF	GGDEF-EAL	EAL	HDGYP
**Total proteins**	**206**	**387**	**122**	**39**
**Localization signals**				
**Without localization signal**	171 (83%)	274 (71%)	120 (98.4%)	39
**With localization signal**	35 (17%)	113 (29%)	2 (1.6%)	0
Transmembrane	29	98	1	0
Sec	6	11	1	0
Tat	0	4	0	0
Lipoprotein	0	0	0	0
**Partner domains**				
**Without partner domains**	120 (58%)	137 (35%)	83 (68%)	16 (41%)
**With partner domains**	86 (42%)	250 (65%)	39 (32%)	23 (59%)
GAF	24	130	3	
PAS	35	62		
Response_reg	12	12	1	23
HDOD	12		27	
Protoglobin	3	80	3	
CZB		62		
MASE		5		
FhlA	9	9		
PRK13558	9	15		
SMC_N	11			
type_I_hlyD	7			
BaeS	2	4		

**FIGURE 5:**
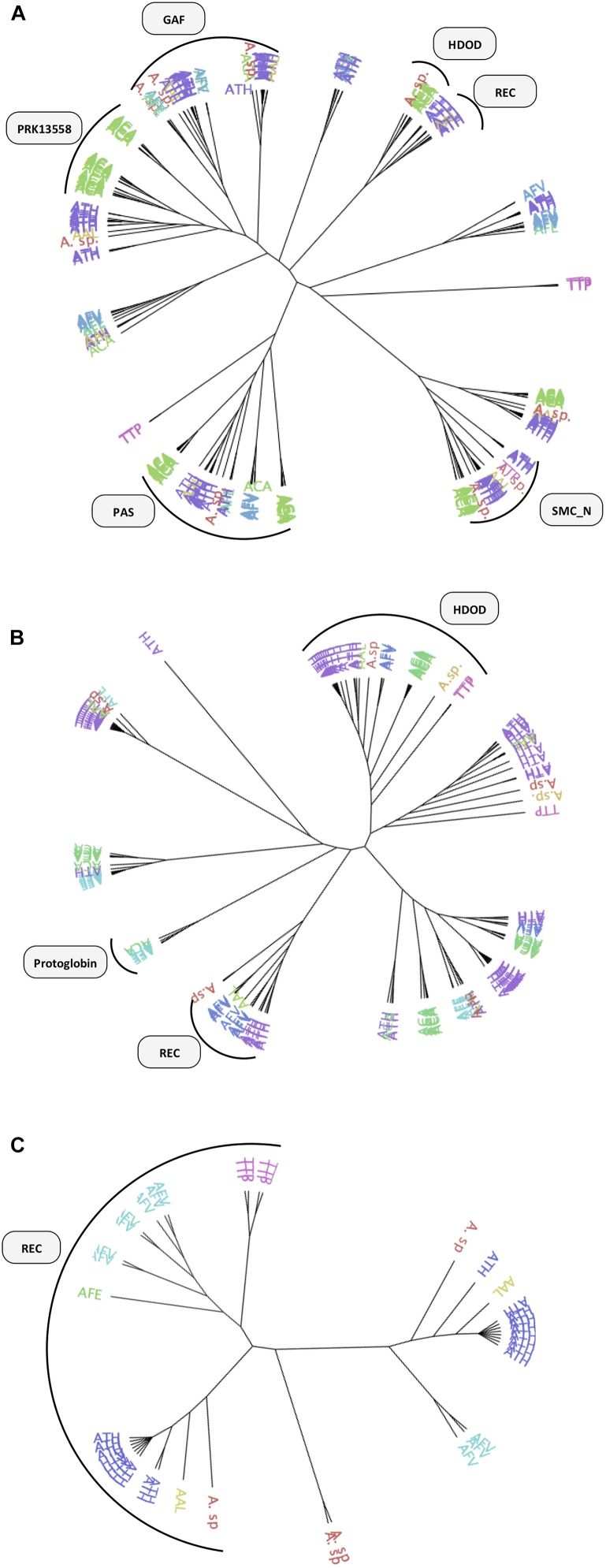
Phylogenetic tree of DGCs and PDEs proteins identified in the acidithiobacilli. **(A)** GGDEF DGCs, **(B)** EAL, and **(C)** HDGYP PDEs. Sensor domains are represented graphically next to the corresponding tree branches. Proteins are colored by taxonomy as follows: TTP, *Thermithiobacillus tepidarius* (pink); ACA, *Acidithiobacillus caldus* (green); ATH, *Acidithiobacillus thiooxidans* (purple); AAL, *Acidithiobacillus albertensis* (yellow); AFV, *Acidithiobacillus ferrivorans* (blue); AFE, *Acidithiobacillus ferrooxidans* (light blue).

The *Acidithiobacillus* species analyzed encode in their genomes a vast diversity of genes linked to c-di-GMP metabolism (total 754), including opposing diguanylate cyclase (DGCs) and phosphodiesterase (PDEs) activities (Figure 4 and Table 2). The DGC activity resides in the GGDEF protein domain (pfam00990), while PDE activity resides in two unrelated domains: EAL (pfam00563) and HD-GYP (COG2206). Globally, *Acidithiobacillia* members show a higher proportion of synthesis versus degradation elements, which agrees with reported trends in other microorganisms (Seshasayee et al., 2010; Römling et al., 2013). As it occurs in other microorganisms, both synthesis and degradation functions in the acidithiobacilli take place in different configurations: GGDEF-only (206 in total), GGDEF-EAL combined (387 in total), EAL-only (122 in total), and HD-GYP-only (39 in total). Notably, HD-GYP proteins are present in all species except in the moderate thermophilic bacteria *A. caldus* (Figure 4A, 5C).

C-di-GMP metabolism proteins bare different subcellular localization signals that partition them differentially at inner or outer membrane, periplasmic space or even secreted out of the cell. The outer membrane localization signals are mostly represented in synthesis-related proteins (29% in GGDEF-EAL and 17% in GGDEF-only) rather than degradation related proteins (2% in EAL-only and 0% in HD-GYP) (Table 2). Twenty five percent of all *Acidithiobacillus* GGDEF-EAL proteins have predicted transmembrane regions. Besides, some species present GGDEF-EAL proteins with signals for translocation through internal membrane, which may reach the periplasm remaining able (or not) to be secreted through outer membrane by the type 2 secretion system (T2SS) protein complex (Cianciotto, 2005). Two subgroups can be distinguished, a GCS-GAF-GGDEF-EAL with *tat* signal present in *A. caldus*, and a few GGDEF-EAL proteins with *sec* signal present in *A. albertensis*, *A. ferrivorans*, and *A. thiooxidans.* The last two bacteria also present GGDEF-only proteins with localization signals.

Most GGDEF-EAL proteins (65%) and nearly half of the GGDEF-only proteins (42%) contain partner domains (Table 2). Most common partner domains found to concur with c-di-GMP metabolism proteins in *Acidithiobacillia* are shown in Figure 4B, 5A–C including: PAS (Period circadian protein, Aryl hydrocarbon receptor nuclear translocator protein and Single-minded protein), GAF (cGMP specific phosphodiesterases, Adenylyl cyclases and FhlA), REC (response regulator receiver domain), Protoglobin [or globin coupled sensor (GCS)], and CZB (Chemoreceptor Zn-Binding domain). Some of these domains are very well-characterized and thus their role in signal transduction can be robustly inferred. For example, the REC domain is typically present in the receiver element (or response regulator) of two-component signal transduction systems (Zschiedrich et al., 2016) and undergoes distinctive conformational changes upon phosphorylation (e.g., Lee et al., 2001). Both *A. ferrivorans* SS3 and *A. caldus* ATCC 51756^T^ encode in their genomes a REC-GGDEF-EAL type protein and this gene is preceded by a histidine kinase encoding gene, in a configuration that is characteristic of well-known two-component systems. In the case of *A. caldus*, this two-component system is genetically linked to other genes involved in the SOS response, suggesting that this nucleotide dependent signal transduction pathway may trigger a collective behavior such as biofilm formation in response to DNA damaging conditions, as recently demonstrated in *Pseudomonas aeruginosa* (Weitao, 2011). On the other hand, the roles of other sensor domains are not so easy to relate to particular input signals (and/or output responses). This is the case of the small molecule-sensing domains, PAS and GAF, were significant sequence variability has been reported to occur (Zoraghi et al., 2004; Gilles-González and González, 2005). Just a few c-di-GMP metabolism proteins containing GAF domain have been characterized so far. Lcd1 (GAF-GGDEF) protein from *Leptospira interrogans* increase its DGC activity upon cAMP binding to its GAF domain (da Costa Vasconcelos et al., 2017). Recently, it was demonstrated that a bifunctional enzyme (GAF-GGDEF-EAL) from *Mycobacterium smegmatis* binds GDP at GAF domain, increasing PDE activity and decreasing DGC activity, which causes a quick decrease of intracellular levels of c-di-GMP (Chen et al., 2018). Thus, the GAF domain may establish a functional link between small nucleotides and c-di-GMP signaling. PAS and GAF domains share a similar structural fold, but are distantly related (Aravind and Ponting, 1997; Ho et al., 2000). Using diverse prosthetic groups, such FAD, heme and biliverdin, PAS domains bind different signals such as redox potential (Qi et al., 2009), O_2_ (Chang et al., 2001; Tuckerman et al., 2009), and light (Tarutina et al., 2006). To elucidate which signals may be transduced by PAS domains found in *Acidithiobacillia* acidophiles, we compared the DGCs derived PAS domains with experimentally validated representatives from other bacterial species. Most *Acidithiobacillus* DGCs and PDEs PAS-domains grouped with molecular oxygen and redox potential sensing referenced domains (Supplementary Figure S1F). This seems relevant in the context of the oxygen, redox and energetic requirements of the acidithiobacilli (Nitschke and Bonnefoy, 2016).

Together, these numbers suggest that *Acidithiobacillus* spp. are able to transduce several signals, originated both extra-and intra-cellularly, in c-di-GMP synthesis, while its degradation may be regulated in a different way. *Acidithiobacillus* PDEs are less likely to be regulated by externals cues given the low diversity of accessory domains identified. Near 70% of the EAL proteins containing accessory domains correspond to HD-related output domain (HDOD; EAL-HDOD configuration) (Figure 5B), which has been involved in the indirect activation of the transcription of chemotaxis genes (Cha et al., 2019). On the other hand, 100% of HDGYP proteins containing accessory domains correspond to the receiver element of two-component signal transduction systems [REC-HDGYP configuration (59% of total HD-GYP proteins)]. In these cases, the output of the two-component system may be the degradation of c-di-GMP. The rest of EAL and HDGYP PDEs are probably regulated at transcriptional level.

The abundance and diversity of c-di-GMP metabolism enzymes is thought to reflect the extent and complexity of the c-di-GMP dependent network in any given microorganism. In this respect, we observed important differences between *Acidithiobacillus* species (Figure 4A and Supplementary Table S3), in both total and relative numbers of c-di-GMP metabolism elements (DGCs and/or PDEs): 34-29 for *A. thiooxidans* > 29 for *A. albertensis* and *T. tepidarius* > 22-16 for *A. caldus* > 28-10 for *A. ferrivorans* > 8-4 for *A. ferrooxidans*. As a whole, these numbers show no correlation with the genome size of each strain analyzed (data not shown). The highest net number of DGCs/PDEs observed occurred in *A. thiooxidans* GD1-3, with a total of 34 c-di-GMP metabolism elements predicted. In turn, the lowest number occurred in *A. ferrooxidans* strains. The type of strains of the *A. ferrooxidans*, ATCC 23270^T^ and strain ATCC 53993, had only four predicted c-di-GMP metabolism proteins, the lowest number in the whole *Acidithiobacillia* class, potentially representing the minimal essential set of DGCs/PDEs for the taxon. Yet, only one of them is conserved through the *Acidithiobacillia* class members (AFE_1712, Protoglobin-GGDEF-EAL-CZB), and two other across *A. ferrivorans* strains (AFE_3057, GAF-PAS-GGDEF-EAL, and AFE_1725, PAS-GGDEF-EAL-CZB). The fourth gene (AFE_1707, GAF-PAS-PAS-GAF-GGDEF-EAL) is shared only with *A. thiooxidans*. This distribution suggests that DGCs/PDEs shared with specific groups may have been gained in informative ecological contexts (e.g., through acquisition of gene islands) that may provide clues on the specific effectors targeted by these and/or other specific control proteins.

The larger the number of transducing components, the larger the range of potential signals that may be integrated and the number of cellular functions that may be expected to be targeted. For instance, host-associated bacteria have few c-di-GMP metabolism proteins while free-living organisms as a general rule tend to have more (Seshasayee et al., 2010). In this respect it is interesting to understand why *A. ferrooxidans*, one of the more versatile representatives of the taxon [capable of aerobic growth using ferrous iron, several different RISCS and hydrogen as electron donor and facultative ferric iron reduction also in the absence of oxygen (Quatrini and Johnson, 2018)], has the fewest. This observation stands for both culture collection and recent isolates of the species. Alternative regulation pathways (that partially replace regulatory function of c-di-GMP) could be part of the explanation. It has been demonstrated that *A. ferrooxidans* possesses a functional Quorum Sensing type AI-1 system (Farah et al., 2005), which is absent in other *Acidithiobacillus* (Valdés et al., 2008; this study, data not shown). Yet, additional underlying reasons require further investigation of the nature of the control modules, the number and diversity of targeted effectors and the interactions established by the elements of this system, both of which are quite challenging tasks.

#### A Plethora of c-di-GMP Binding Effector Proteins Are Scattered Along the Genome

The c-di-GMP second messenger has been proven to bind a wide diversity of effector proteins triggering a plethora of different specific physiological and behavioral responses (Chou and Galperin, 2016). This diversity is also represented in c-di-GMP effectors predicted in the genomes of sequenced *Acidithiobacillia* representatives (Supplementary Table S3). These include PilZ domains, PelD and FleQ proteins, and other less characterized proteins such as CckA, MshE, CX_3703, BdcA, BldD, VpsT, BcsE, BCAM1349, Clp, BrlR, LtmA, VpsR, and PnpA (not shown).

**FIGURE 6:**
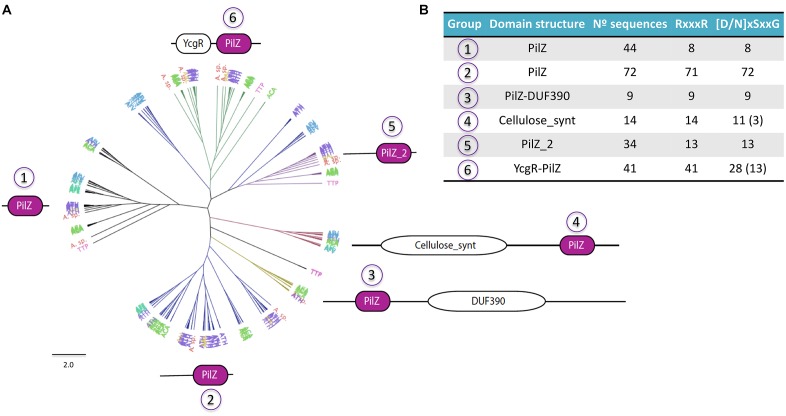
Diversity of *Acidithiobacillia* PilZ proteins. **(A)** Phylogenetic tree showing the six principal groups of PilZ proteins found in the acidithiobacilli. **(B)** Conservation of signature motifs for c-di-GMP binding in PilZ protein groups.

Among these, the PilZ protein domain is considered to be the universal c-di-GMP-binding adaptor and experimental demonstrations of this fact abound in the literature (Ryjenkov et al., 2006; Boehm et al., 2010; Fang and Gomelsky, 2010; Paul et al., 2010; Wilksch et al., 2011). Every *Acidithiobacillus* genome analyzed encodes PilZ proteins. In agreement with the low number of c-di-GMP metabolism proteins, *A. ferrooxidans* strains showed the fewest amount of PilZ domains too. As occurs in other bacteria, the PilZ domain exists alone or in conjunction with other domains in the same polypeptide chain in the acidithiobacilli predicted proteins, suggesting that binding of c-di-GMP can trigger different responses in this acidophilic taxon as well. A phylogenetic tree of the PilZ family proteins identified in the acidithiobacilli shows six large protein groups clearly differentiated by their domain configuration (Figure 6A). Most of PilZ proteins of *Acidithiobacillus* (65.4%) are predicted to contain just the PilZ domain, however, they could be distributed at least in three different sub-groups (groups 1, 2, and 5), which differ in their ability to bind c-di-GMP due the presence/absence of two sequence motifs: RxxxR and [N/D]xSxxG (Figure 6B and Supplementary Figure S1G). These motifs are separated by ∼20 amino acid residues, and each one mediates the protein interaction with a guanine base of the cyclic di-nucleotide molecule (Benach et al., 2007; Ko et al., 2010; Habazettl et al., 2011). PilZ-only proteins are generally related with Type IV Pili (T4P) formation and twitching motility, which has been implicated in irreversible attachment to surfaces, microcolony grouping and structural development of biofilm (O’Toole and Kolter, 1998 Semmler et al., 1999). PilZ proteins of Group 3 consist in long proteins (∼1000 residues) that coupled PilZ domain with uncharacterized domain DUF390, and they are present only in *A. caldus* strains. Group 4 are conformed by proteins containing Cellulose_synt domain (pfam03552), which are homologs to bacterial cellulose synthase A subunit (BcsA), and therefore may synthetize cellulose or some other glucose-based exopolysacharrides needed for attachment and extracellular matrix construction. In *Komagataeibacter xylinus*, cellulose synthesis depends on the presence of the cellulose synthase complex (Saxena et al., 1994), which binds c-di-GMP directly (Weinhouse et al., 1997) and increases 200 times its basal activity (Ross et al., 1985). Finally, group 6 contain proteins with an YcgR domain, known to down regulate flagellar motility in response to c-di-GMP binding (Fang and Gomelsky, 2010; Paul et al., 2010).

**Table 3:** c-di-GMP elements in Mobile Genetic Elements present in *Acidithiobacillia* closed genomes.

Strains	MGE/plasmid	Metabolism	Effector	Targets
*A. caldus* ATCC 51756	ICEAca_TY_2^∗^	PAS-GGDEF-EAL	PilZ	T4P
		EAL	FleQ	Conjugation system
			YcgR-PilZ	Flagella
	ISR7	GGDEF		
		GGDEF		
		GGDEF		
		EAL		
		GGDEF		
	pAca1^∗^	GCS-GGDEF-EAL	PilZ	Cytochromes, Relaxase
	mpAca1	N.D.	PilZ	Cellulose synthase
*A. caldus* SM-1	ICEAca_SM1_1^∗^	PAS-GGDEF-EAL	PilZ	Chemotaxis system
		GCS-EAL		Conjugation system
				
	ISR	GGDEF		
		GGDEF		
		PAS-GGDEF-EAL		
		EAL		
		PAS-PASGGDEF		
*A. ferrooxidans* ATCC 23270	ICEafe_23270_2^∗^	GAF-PAS-PAS-GAF-GGDEF-EAL	PilZ	Conjugation system
		Protoglobin-GGDEF-EAL-CZB	FleQ_like	Regulation of transcription
		PAS-GGDEF-EAL-CZB	GTP-Binding protein	
*A. ferrooxidans* ATCC 53993	ICEafe_53993_2^∗^	GAF-PAS-PAS-GAF-GGDEF-EAL	PilZ	Conjugation system
		Protoglobin-GGDEF-EAL-CZB		
		PAS-GGDEF-EAL-CZB		
*A. ferrivorans*	ICEAfv_SS3_1	GGDEF	N.D.	??
		GGDEF-EAL		

*N.D., non-detected. ^∗^MGE/plasmid containing complete c-di-GMP control modules.*

Another class of c-di-GMP effector protein was widely found in *Acidithiobacillia*. FleQ is a protein composed of three domains (FleQ-AAA-HTH) involved in the transcriptional regulation of flagella and EPS production. In *P. aeruginosa*, the binding of c-di-GMP in the AAA domain of FleQ determines a slight decrease in the transcription of flagellar genes, but simultaneously causes a great increase in the transcription of the *pelABCDEFG* operon required for the synthesis of PEL exopolysaccharide and the formation of biofilms (Hickman and Harwood, 2008). Some of FleQ proteins of *Acidithiobacillus* do not possess the FleQ domain, however, they present most of the key amino acids for the binding of c-di-GMP in their AAA domains (Supplementary Figure S1H).

The *pel*-like operon was also found in *Acidithiobacillus* strains. While bacterial cellulose synthase machinery is present in both sulfur and sulfur/iron oxidizer species, a complete *pel*-like operon is only present in *A. caldus, A. thiooxidans*, and *Acidithiobacillus* sp. SH (Supplementary Table S3), corroborating its presence only in sulfur-oxidizing species (Castro et al., 2015; Díaz et al., 2018). This operon encodes the c-di-GMP receptor protein PelD, which contains the key amino acid for c-di-GMP binding (Supplementary Figure S1I). By comparing wild type and Δ*pelD* null-mutant strains, Díaz et al. (2018) have recently reported that PelD and c-di-GMP pathway are involved in biofilm formation and architecture in *A. thioooxidans*. This is likely also true for *A. caldus*, and *Acidithiobacillus* sp. SH. The absence of the *pel*-like operon in *A. albertensis* is intriguing due to its close phylogenetic relationship with *A. thiooxidans* (Castro et al., 2017).

In addition to the typical effector proteins, catalytically degenerate and inactive GGDEF and EAL domains have been shown to act as effector proteins by allosteric binding of c-di-GMP (Beyhan et al., 2008; Abel et al., 2011; Newell et al., 2011; Qi et al., 2011). Candidate effector proteins were also identified in the *Acidithiobacillia* class among the predicted DGCs and PDEs, but their function as such awaits experimental validation.

**FIGURE 7:**
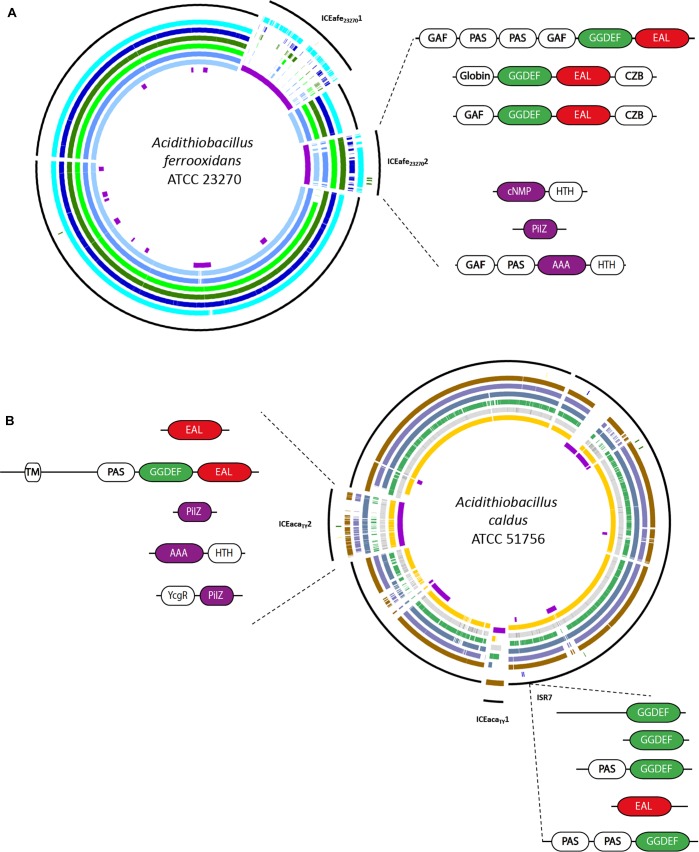
*Acidithiobacillus* mobile genetic elements (MGEs) containing c-di-GMP related proteins. **(A)** MGEs present in *A. ferrooxidans* ATCC 23270^T^. Genomes order: protein predictions in AFE ATCC 23270, AFE Wenelen, AFE DLC-5, AFE GGI-221, AFE 53993, AFE HEL-18, AFE YQH-1, MGE predictions. **(B)** MGEs present in *A. caldus* ATCC 51756^T^. Genomes order: protein predictions in ACA ATCC 51756, ACA SM-1, ACA MTH-04, ACA DX, ACA S1, ACA ZBY, ACA ZJ. MGE predictions. C-di-GMP related protein domain configurations are detailed.

#### C-di-GMP Circuits in Mobile Genetic Elements (MGEs) as a Means to Infer Probable Output Phenotypes

Inside the cell, effector molecules interact with cognate target components to produce a variety of output phenotypes. However, given the multiplicity of c-di-GMP related genes and their scattered distribution within most bacterial genomes, the elements that belong to the same c-di-GMP control module are difficult to uncover. Furthermore, since many of the predicted protein products with GGDEF/EAL domains pertain to the flexible genome of a given species [e.g., 40% of the DGCs/PDEs of *A. albertensis*^T^ are exclusive to this strain (Castro et al., 2017)], concurrencies between cyclases, esterases, effectors and targets are blurry. Previous work from our group and others has shown that the *Acidithiobacillus* spp. harbor different types of MGEs including prophages (Tapia et al., 2012; Covarrubias et al., 2018), plasmids (reviewed in Quatrini et al., 2016) and integrative conjugative elements (ICEs) (Bustamante et al., 2012; Acuña et al., 2013). Albeit a few reports on the occurrence of c-di-GMP pathway modules on MGEs exist (Bordeleau et al., 2010; Madsen et al., 2018), bioinformatic analyses of several *Acidithiobacillus* genomes point out the frequent occurrence of c-di-GMP related elements in this taxon’s mobilome.

**FIGURE 8:**
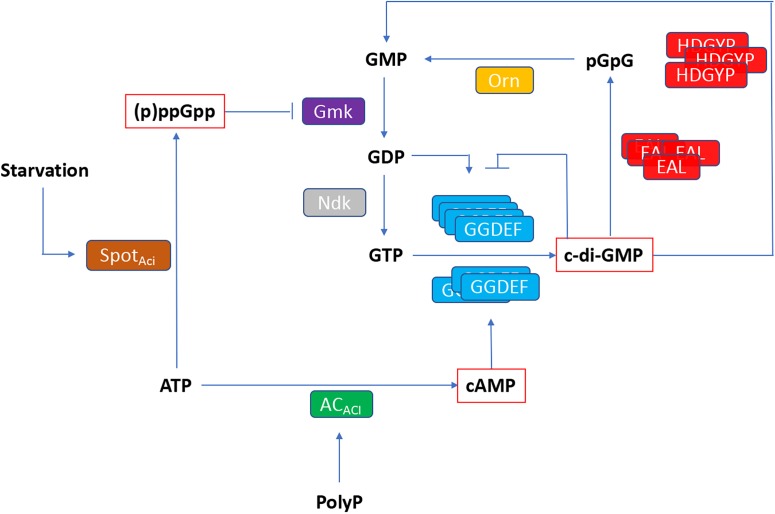
Working model of relationship between (p)ppGpp, cAMP and c-di-GMP based second messenger signaling in *Acidithiobacillus*. SpoT_Aci_, bifunctional (p)ppGpp synthetase/guanosine-3′-5′-*bis*(diphosphate) 3′pyrophos′hohydrolase; AC_Aci_, adenylyl cyclase; Ndk, nucleoside diphosphate kinase; Gmk, GMP kinase; GGDEF, DGC domain; EAL and HDGYP, PDE domains; Orn, oligoribonuclease.

Considering that MGEs behave as cohesive heritable units and that genes present on a particular element or replicon (in the case of plasmids) are likely to have co-evolved to accomplish a common function, we decided to inspect this aspect in detail as a strategy to uncover potential c-di-GMP responsive control modules. Using genome information and comparative genomics tools, we mapped c-di-GMP related elements (DGC, PDE, c-di-GMP effector proteins) to the core and flexible/mobile genome of five completely sequenced *Acidithiobacillia* members: *A. caldus* ATCC 51756^T^, *A. caldus* SM-1, *A ferrooxidans* ATCC 23270^T^, *A. ferrooxidans* ATCC53993, and *A. ferrivorans* SS3. For every candidate gene identified, systematic analyses of the genomic context were performed in order to derive probable target components through functional association analysis. Results obtained are summarized in Table 3. All five microorganisms analyzed harbored at least one integrated MGE and/or a plasmid baring one or more signature proteins for c-di-GMP metabolism. In most of these elements, one or more potential targets could be pinpointed (Table 3). Concurrence of c-di-GMP synthesis/degradation genes, effectors genes and either biofilm- or conjugation-related genes was observed in four of out five genomes alluded.

In the case of *A. ferrooxidans* ATCC 23270 (Figure 7A), both previously identified ICE elements ICE*Afe*1 (Bustamante et al., 2012) and ICE*Afe*2 (Bustamante et al., 2014) code for DGCs and/or PDEs. One of these elements, ICE*Afe*2, which encodes the carboxysome protein complex and the RuBisCo biphosphate carboxylase enzyme, both relevant for CO_2_ fixation, is partially conserved in *A. ferrooxidans* ATCC 53993. This element also bares three GGDEF-EAL proteins with accessory domains of the GAF, PAS, and GCS type in the vicinity of a PilZ-type effector proteins and *trb*-type conjugative genes, suggesting a connection between these c-di-GMP dependent signal transduction pathways and conjugation. In *A. caldus* ATCC 51756^T^ (Figure 7B) the ICE*Aca*_TY_.2, a 180 kb MGE (Acuña et al., 2013), comprises a predicted bifunctional DGC/PDE (Aca1879) and the c-di-GMP receptors/effector proteins PilZ (Aca1908), FleQ (Aca1947) and YcgR-like (un-annotated gene between Aca1939 and Aca1940). In the immediate context of *pilZ*, chemotaxis and flagellar motility related functions were identified. Also, in the vicinity of the flagellar biosynthesis genes, we identified the Aca1947 gene, whose genetic product share is 69% of similarity with FleQ, a well-characterized transcriptional activator of the flagellar apparatus gene operon in other microorganisms (Arora et al., 1997). The third predicted effector protein identified in the *A. caldus* ICEAca_TY_.2, YcgR-like, shows 36% of similarity with flagellar brake protein YcgR. In enterobacteria the YcgR protein affects negatively both swimming and swarming in a c-di-GMP dependent manner (Ryjenkov et al., 2006; Christen et al., 2007). An equivalent or related function may be proposed for the *ycgR*-like gene present in ICE*Aca*_TY_.2, although demonstration of this function remains a challenge. Besides, a region rich in predicted insertion sequences (IS) was identified in the *A. caldus* ATCC 51756^T^ chromosome (and conserved in a SM1), which contains five predicted c-di-GMP elements (3 GGDEF, 1 inactive GGDEF and 1 EAL). One of the GGDEF proteins encoded by genes present in this c-di-GMP “hot spot” (designated ISR7, for IS-rich region), Aca1319 (formerly Aca1413), has already been experimentally characterized (Castro et al., 2015). This protein showed to be a functional DGC, responsible for the synthesis of most c-di-GMP produced by *A. caldus* when grown under standard conditions with elemental sulfur as energy source. Moreover, its gene has been mutated (ΔAca1319), being one of the few *Acidithiobacillus* mutants obtained world-wide (Castro et al., 2015). In comparison with the wild type strain, this mutant presented delayed attachment to a solid substrate in early stages of mineral colonization, and also exacerbated swarming motility levels. Also, putative c-di-GMP control modules were identified in two out of three *A. caldus* ATCC 51756^T^ plasmids, including a *pilZ* ortholog occurring next to the cellulose synthetase in the mp*Aca*1 megaplasmid. These results point to an important contribution of the mobile genetic complement in nucleotide signal transduction in these acidophiles.

### Extracellular Nucleotide Signaling in Acidic Environments?

Several of the c-di-GMP metabolism proteins identified in this study are candidate integral membrane proteins, others are predicted to be targeted to the outer membrane and yet others are presumably secreted into extracellular media (Table 2). Most proteins located in the extracellular compartments were GGDEF (17%) and GGDEF-EAL combined proteins (29%) and these occurred invariably in all *Acidithiobacillus* species analyzed. Such a finding raises the possibility that c-di-GMP may act as an extracellular signal linking perception of specific environmental or populational cues, to specific alterations in cellular function. In other bacteria, extracellular c-di-GMP has been shown to inhibit adherence of *Streptococcus mutans* to tooth surfaces (Yan et al., 2010), while treatment of *Staphylococcus aureus* with external c-di-GMP inhibited cell adhesion in liquid medium, and adherence to human epithelial cells (Karaolis et al., 2005). Certainly, harsh conditions imposed by an acidic milieu could prevent extracellular communication through nucleotides. However, their stability at acidic condition may be robust enough for message delivery; c-di-GMP shows resistance to boiling and to extreme acid exposition (Karaolis et al., 2005). Cyclic AMP and c-di-AMP are good candidates for this kind of signaling, since they also are stable under low pH (and high temperature). The half-life of cAMP is close to 30 min at 100°C in 1N HCl (Revankar and Robins, 1982), while c-di-AMP shows slow degradation into different intermediaries at 90°C and pH below 3 (Ora et al., 2012).

Bioinformatic analysis performed in this study failed to identify proteins carrying the domains required for c-di-AMP synthesis (DisA_N, pfam02457; Witte et al., 2008; Bai et al., 2012; Corrigan and Gründling, 2013) in the *Acidithiobacillia*, but hallmark proteins responsible for c-di-AMP hydrolysis (DHH domains, pfam01368; Rao et al., 2010) and candidate effectors proteins (KtrA, KdpD) are present in most genomes analyzed (Supplementary Figure 1J). Interestingly, other acidophilic bacteria (e.g., *Leptospirillum* and *Sulfobacillus* among others) do possess all genes required to construct a functional c-di-AMP signaling pathway, including synthesis, degradation and effectors proteins (data not shown). Therefore, evidence gathered in this work suggests that extracellular signal transduction pathways relying on cyclic nucleotidic second messengers may indeed exist in acidic econiches and raises the intriguing possibility that these nucleotides may mediate both intra- and inter-species communication. The existence and role of these interaction merits further exploration.

## Conclusion

This study is the first comprehensive genomic analysis of nucleotide second messengers-based signaling pathways in polyextremophilic bacteria belonging to the *Acidithiobacillia* class. Our results showed that these acidophiles conserve at least three signaling mechanisms based on (p)ppGpp, cAMP, and c-di-GMP. As in other bacteria, in *Acidithiobacillus* the (p)ppGpp synthesis through SpoT_ACI_ is predicted to respond to nutrient limitation, reprograming global gene expression through direct binding to RpoZ RNA polymerase subunit. Cyclic AMP nucleotide synthesis in the acidithiobacilli seems to be limited by ATP availability, yet functional association-based inference suggests it could also be regulated by the presence of polyP. To our knowledge, this link has not been pointed out nor explored previously in the acidithiobacilli. The most versatile and diverse signaling pathway uncovered in the *Acidithiobacillia* was the one relying on c-di-GMP. On the basis of diverse partner sensor, synthesis and degradation domains, this pathway is predicted to transduce environmental cues (such as redox potential and oxygen levels) as well as intracellular signals (such as phosphorylation cascades), into differential levels of intracellular c-di-GMP. These, in turn, are predicted to regulate flagellar based motility, substrate attachment, and subsequent biofilm development through binding to diverse effector proteins included PilZ domain, FleQ and PelD proteins encoded in the gene vicinity of the DGCs and/or PDEs or confined to specific MGEs. All the (p)ppGpp, cAMP, and c-di-GMP second messenger systems, may cross-talk with each other through different components of the respective pathways (Figure 8). According to this working model, (p)ppGpp is predicted to have a direct impact on GDP and GTP synthesis, through inhibition of GMK activity. In turn, GTP, GDP, and cAMP levels feed in the c-di-GMP pathway principally through DGCs, while PDEs recycle c-di-GMP to restore nucleotide pools. While GTP is the substrate for c-di-GMP synthesis, GDP and cAMP molecules could be sensed by GGDEF proteins containing GAF domains, regulating its DGC activity.

## Data Availability

Protein alignments of Supplementary Figure S1 can be found in Figshare, https://figshare.com/s/63b7234a67db25ff7a8b; 10.6084/m9.figshare.7553921.

## Author Contributions

MC and RQ conceived and supervised the study and metabolic reconstruction analysis. AM-B and CR-V carried out the sequence processing and bioinformatic analyses. NG and MD contributed in data analysis. MC, AM-B, and RQ analyzed and interpreted the data and wrote the manucript. All authors read and approved the final manuscript.

## Conflict of Interest Statement

The authors declare that the research was conducted in the absence of any commercial or financial relationships that could be construed as a potential conflict of interest.

## Footnotes

1 https://web.expasy.org/compute_pi/

## Supplementary Material

**FIGURE S1.** Supporting alignments for protein domains in nucleotide-driven signal transduction pathways of the acidithiobacilli. **(A)** SpoT; **(B)** RpoZ; **(C)** GMK; **(D)** AC; **(E)** CRP; **(F)** PAS; **(G)** PliZ; **(H)** FleQ; **(I)** PelD; and **(J)** DHH. Figshare, https://figshare.com/s/63b7234a67db25ff7a8b; 10.6084/m9.figshare.7553921.

TABLE S1 *Acidithiobacillia* class genomes analyzed in this study.

TABLE S2 Hallmark genes or profile list used in the analysis.

TABLE S3 Abundance of proteins predicted to be involved in nucleotide second messenger metabolism in different replicons from *Acidithiobacillus*.
